# ASSESSING STAFF PERCEPTIONS OF WORKPLACE SAFETY IN NURSING HOMES: ITEM SET DEVELOPMENT AND TESTING

**DOI:** 10.1093/geroni/igad104.0771

**Published:** 2023-12-21

**Authors:** Katarzyna Zebrak, Naomi Yount, Joann Sorra, Laura Gray, Jessica Kirchner, Joanne Campione

**Affiliations:** Westat, Rockville, Maryland, United States; Westat, Rockville, Maryland, United States; Westat, Westat, Maryland, United States; Westat, Rockville, Maryland, United States; Westat, Rockville, Maryland, United States; Westat, Durham, North Carolina, United States

## Abstract

Workplace safety is important for advancing resident safety and eliminating harm to both nursing home staff and residents. The goal of this study was to develop and test survey items to assess how the organizational culture in nursing homes supports workplace safety for staff. After conducting a literature review and background interviews with nursing home staff and workplace safety experts, we identified important areas of workplace safety culture (i.e., workplace hazards; moving/transferring/lifting residents; inappropriate resident behavior toward staff; interactions among staff; supervisor/management support for workplace safety; workplace safety reporting; and, work stress/burnout) and drafted survey items to assess each area. These draft items were administered with the AHRQ Surveys on Patient Safety Culture (SOPS) Nursing Home Survey to staff in 48 U.S. nursing homes. We conducted psychometric analysis on data from 2,468 respondents. Confirmatory factor analysis results and internal consistency and site-level reliability coefficients were acceptable for the 18 survey items grouped into 6 composite measures. All composite measures were significantly correlated (p< 0.05) with each other and with the overall rating on workplace safety, indicating adequate conceptual convergence among survey measures. On average, 33% of respondents reported symptoms of burnout. Higher burnout was significantly correlated with lower job satisfaction (rs=-0.42) and greater intent to leave the nursing home (rs=0.37). Nursing homes and researchers can use the Workplace Safety Supplemental Item Set to assess nursing home staff burnout and the dimensions of organizational culture that support staff safety, and to identify both strengths and areas for improvement.

